# Clinicopathological significance and prognostic value of E-cadherin expression in non-small cell lung cancer: A protocol for systematic review and meta-analysis

**DOI:** 10.1097/MD.0000000000024748

**Published:** 2021-02-19

**Authors:** Dong Chao, Gawei Hu, Qingxin Li

**Affiliations:** Department of Thoracic Surgery, The 940th Hospital of Joint Logistics Support Force of Chinese People's Liberation Army, Lanzhou, Gansu, China.

**Keywords:** E-cadherin, meta-analysis, non-small Cell Lung Cancer, prognosis

## Abstract

**Background:**

E-cadherin, a calcium-dependent cell adhesion molecule, as an important mediator of adhesion and signaling pathway, plays a key role in maintaining tissue integrity. However, the association of E-cadherin expression with clinicopathological features and prognostic value in non-small cell lung cancer (NSCLC) is still controversial. Therefore, the purpose of the study is to explore the clinicopathological features and prognostic value of E-cadherin expression in non-small cell lung cancer by meta-analysis.

**Methods:**

PubMed, EMBASE, Cochrane Library, and Web of Science were searched to collect the studies about expression of E-cadherin and clinicopathological features and prognosis of non-small cell lung cancer. The last search time was May 2020. Stata 15.0 software was used for statistical analysis.

**Results:**

A total of 35 studies were included, of which the results showed that high expression of E-cadherin compared with its low expression, for overall survival, HR = 0.68 (95% CI:0.64–0.73, *P *< .05); for disease-free survival or progression-free survival, HR = 0.54 (95% CI: 0.44–0.67); low differentiation of lung cancer compared with moderate and high differentiation, OR = 0.40 (95% CI: 0.27–0.58, *P *< .05); Advanced lung cancer compared with early stage, OR = 0.54 (95% CI: 0.44–0.66, *P *< .05); lymph node metastasis compared with non-lymph node metastasis, OR = 0.49 (95% CI: 0.31∼0.77).

**Conclusion:**

Low expression of E-cadherin is closely related to poor prognosis of patients with NSCLC, promoting tumor staging and lymph node metastasis, inhibiting tumor differentiation as well.

## 1 Introduction

Along with social progress, environmental pollution has been becoming increasingly serious. Various types of carcinogenic factors are increasing, and incidence of malignant tumors is also increasing. According to statistics, the incidence and mortality of lung cancer ranks first in 2018 global cancer statistics.^[1]^ Among them, the incidence of non-small cell lung cancer accounts for more than 80% of all lung cancers.^[2]^ Thanks to improvement of public health awareness and examination methods, the detection rate of early lung cancer is higher than before. However, most of the early clinical manifestations of lung cancer are mainly cough, which is difficult to pay attention to. Most patients with lung cancer were diagnosed in middle and late stage, who had missed the opportunity of surgical resection. The 5-year survival rate of lung cancer is low, about 15%, which is also easy to relapse.^[3]^ At present, the treatment of non-small cell lung cancer includes surgery, radiotherapy and chemotherapy, targeted therapy, immunotherapy and so on. In recent years, with the introduction of the theory of “precision medicine,” targeted therapy has been paid more attention. A large number of new driving genes have been discovered, and corresponding targeted drugs have come out. For patients with advanced lung cancer, it is one of the most important factors affecting prognosis whether they have driver gene or not and whether they receive targeted therapy or not. The median survival time of patients who have advanced lung cancer with positive driving gene and use targeted therapy is 3.5 years; the median survival time of patients who have negative driving gene and do not receive targeted therapy is 2.4 years; the median survival time of patients who have advanced lung cancer with negative driving gene is only 2.1 years.^[4]^

The internal balance of body and stability of organ function depends on the interaction between cells, and between cells and external environment. Regulate cells phenotype and behavior through adhesion and signal transduction. The molecular basis of cell adhesion is that adhesion molecular receptors mediate the process of recognition and specific binding between cell surfaces and between cells and extracellular matrix ligands. E-cadherin, as a calcium-dependent homo-type cell adhesion molecule, is a transmembrane glycoprotein distributed in all epithelial tissues, which is closely related to the occurrence, invasion and metastasis of cancer.^[5]^ It can promote the adhesion between epithelial cells and maintain the integrity of tissue structure, which is an inhibitory factor of tumor metastasis. The decrease or loss of its expression will weaken the adhesion between tumor cells, which makes them fall off and migrate easily, and lead to tumor invasion and metastasis.^[6]^

At present, there are many studies on the clinicopathological features and prognosis of E-cadherin and non-small cell lung cancer, but the results are not consistent. It has been reported^[7,8]^ that low expression of E-cadherin is not conducive to prognosis of patients with non-small cell lung cancer. However, some studies^[9,10]^ suggested that expression of E-cadherin has nothing to do with prognosis of patients with non-small cell lung cancer. Yang et al.^[11]^ reported that high expression of E-cadherin is beneficial to differentiation of lung cancer. However, the study of Deeb et al^[7]^ suggested that expression of E-cadherin has nothing to do with differentiation of lung cancer. Therefore, this study used a meta-analysis method to comprehensively analyze previous studies on the relationship between E-cadherin expression and clinicopathological features and prognosis of non-small cell lung cancer, in order to provide evidence-based medicine for targeted therapy in non-small cell lung cancer.

## 2 Methods

### 2.1 Document retrieval strategy

PubMed, EMBASE, Cochrane Library, and Web of Science were searched to collect studies upon the correlation between expression of E-cadherin and clinicopathological features and prognosis in non-small cell lung cancer. The retrieval time was from the establishment of databases to May 2020. The search formula was as follows: (“lung cancer” OR “lung neoplasm” OR “lung tumor” OR “lung carcinoma” OR “Non-small cell lung cancer” OR “NSCLC”) and (“E-cadherin” OR “CDH1”) and (“prognosis”). The language was limited to English.

### 2.2 Inclusion and exclusion criteria

#### 2.2.1 Inclusion criteria

1. patients were pathologically diagnosed as non-small cell lung cancer;
2. the study population was divided into high expression group and low expression group of E-cadherin, and the detection method was immunohistochemistry (IHC);
3. full-text articles were published in English;
4. sufficient information was provided to estimate the hazard ratio (HR) or odds ratio (OR) and its 95% confidence interval (CI);
5. correlation between E-cadherin expression and overall survival (OS) and disease-free survival (DFS)/progression-free survival (PFS) was evaluated;

#### 2.2.2 Exclusion criteria

1. patients were followed up for less than 3 years;
2. studies did not focus on humans;
3. if the same author or the same medical center had repeated data, the articles with higher influencing factors and complete data were selected;
4. the Newcastle-Ottawa Scale (NOS) score was less than 6.

### 2.3 Data extraction

Two researchers independently examined the title and abstract of an article to determine whether they should get the full text. The article will be excluded, if it is found that it does not meet the inclusion criteria, after its full text is read through. Whenever there are disagreements, the 2 researchers should resolve them through discussion. A third researcher should be involved when the 2 researchers fail to reach a consensus. The following prognostic information was extracted from the study: author, year of publication, country of the studied population, number of patients, follow-up time, detection method, critical value, HR and its 95% CI. In addition to the basic information and prognosis, regarding the clinicopathological features, the following data were also extracted, such as the number of cases with high and low expression of E-cadherin in TNM stage, tumor grade and lymph node metastasis, and calculated the corresponding OR value and its 95% confidence interval.

### 2.4 Quality evaluation

The quality of the literature was evaluated according to NOS.^[12]^ The literature with less than 6 stars was of low quality, while the literature with 6 stars or above was of high quality. Only those with an evaluation of more than 6 stars were included in this study. Two evaluators assessed literatures independently by cross-checking and reached a consensus through discussion when there were disagreements.

### 2.5 Statistical methods

The data were analyzed by Stata 15.0 statistical software. The relationship between expression of E-cadherin and prognosis of NSCLC was evaluated by HR. If HR of overall survival was not given in the original text, Engauge Digitizer software was used to extract the survival curve data, and then calculate the HR and its 95%CI. OR was used to assess the relationship between expression of E-cadherin and tumor stage, grade and lymph node metastasis of non-small cell lung cancer. The heterogeneity among studies was tested by Q-test. If *I*^2^ ≥ 50%, or *P* ≤ .05, there was heterogeneity among studies, then a random-effects model (REM) was used; if *I*^2^ < 50%, and *P* > .05, there was no heterogeneity among studies, and a fixed-effects model (FEM) was adopted. Publication bias was assessed by Funnel plot and Egger Test. If funnel plot was asymmetric, there was likely to be publication bias. If there was heterogeneity in the aspect of prognosis, a subgroup analysis of ethnicity and tumor stage was conducted to explore the source of heterogeneity.

## 3 Results

### 3.1 Results of literature retrieval

**Figure 1:**
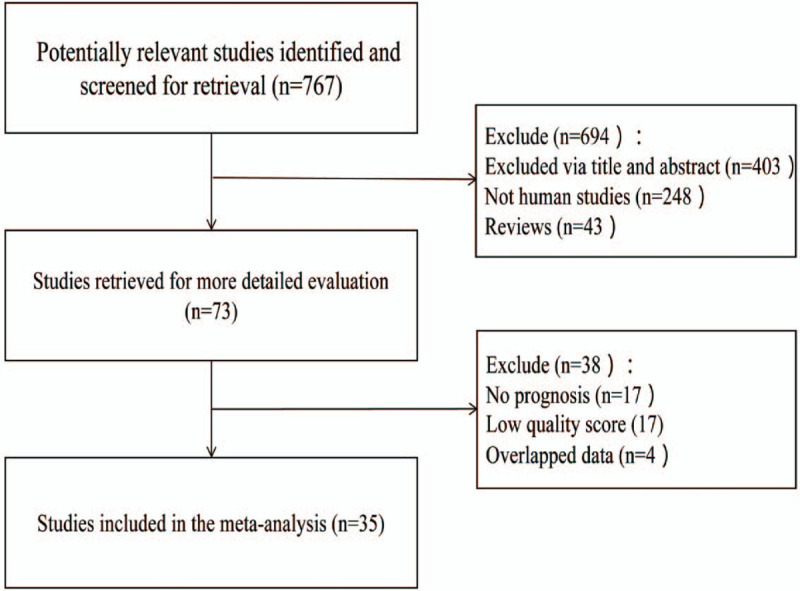
Document retrieval flow diagram.

After a comprehensive search of each database, 35 studies were incorporated into^[7–11,13–41]^ this meta-analysis, including 6744 patients with NSCLC, of whom 2940 had positive expression of E-cadherin. The positive expression rate was 43.6%. More information on the screening process is shown in Figure 1. The basic characteristics and NOS scores of the included literatures are found in Table 1.

**Table 1:** Basic characteristics of the included studies.

First Author	Year	Country	Patients	Tumor stage	Methods of detection	Cut-off value	follow-up (year)	Outcomes	NOS score
Sulzer	1998	Netherland	111	I-III	IHC	10%	>3	①④	8
Kase	2000	Japan	331	I-IV	IHC	70%	>3	①②③④	8
Kimura	2000	Japan	135	I-IV	IHC	80%	>3	①②③④	8
Lee YC	2000	China	207	I-III	IHC	40%	>3	①②③④	8
Liu D	2001	Japan	109	I-IV	IHC	50%	>3	①②③④	8
Hirata	2001	Japan	249	I-IIIA	IHC	10%	>3	①②	8
choi	2003	Korea	141	I	IHC	50%	>3	①②	8
Deeb	2004	USA	118	I-IIIA	IHC	10%	>3	①②	8
Huang C	2005	Japan	173	I-III	IHC	50%	>3	①③④	8
Tamura	2005	Japan	131	I-IIIA	IHC	75%	>3	①②③	8
Saad	2008	Norway	321	I-IIIA	IHC	50%	>3	①	8
Cho	2008	Korea	55	I	IHC	25%	>3	⑤	7
Ruiz	2009	USA	199	I-III	IHC	50%	>3	①	8
Zhu ZH	2009	China	148	IB	IHC	ROC	>3	①	7
Yamashita	2010	Japan	117	I-III	IHC	70%	>3	①	8
Lin Q	2010	China	185	I	IHC	50%	>3	①	7
Sterlacci	2010	Austria	405	I-IV	IHC	25%	>3	①	8
Ono	2010	Japan	107	I	IHC	70%	>3	①	7
Yu J a	2011	China	44	I-IV	IHC	75%	>3	①	8
Yu J b	2011	China	57	I-IV	IHC	75%	>3	①	8
Nagathihalli	2012	USA	284	I-IV	IHC	NR	>3	①	7
Wu SW	2012	China	50	I-III	IHC	score = 1	>3	①②④	8
Feng J	2012	China	103	I-IV	IHC	10%	>3	①	8
Richardson	2012	USA	38	I-IV	IHC	10%	>3	⑥	8
Zhang H	2013	China	204	I-IIIA	IHC	80%	>3	①②③④⑤	8
Kim	2013	Korea	193	I-III	IHC	score = 100	>3	①	8
Zhang X	2013	China	118	I-IIIA	IHC	66%	>3	①	7
Zhao C	2013	China	119	I-IV	IHC	10%	>3	①	8
Hao LG	2014	China	102	I-III	IHC	NR	>3	①⑤	7
Zhou YJ	2016	China	153	I-IV	IHC	50%	>3	①②③⑥	8
Xiong D	2017	China	208	I-IV	IHC	50%	>3	①②③④	8
Li JC	2017	China	134	I-III	IHC	50%	>3	①	8
Yang ZY	2017	China	186	I-IV	IHC	50%	>3	①②③⑥	8
Wang GH	2018	China	78	I-IV	IHC	50%	>3	①②	8
Ci HF	2018	China	163	I-IIIA	IHC	50%	>3	①②③④	8
Imai	2018	Japan	1268	I-IV	IHC	25%	>3	①⑤	8

### 3.2 Results of meta-analysis

#### 3.2.1 Relationship between expression of E-cadherin and prognosis of NSCLC

**Figure 2:**
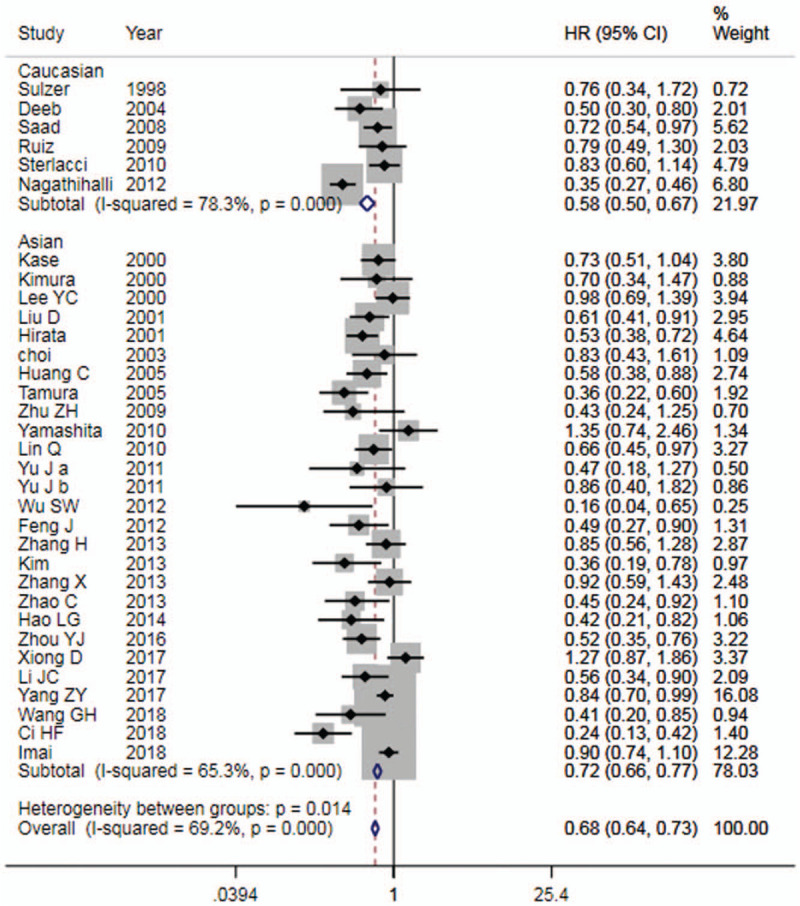
Forest plot of E-cadherin expression and OS in patients with NSCLC. OS = overall survival, NSCLC = non-small cell lung cancer.

All the literatures were included in the analysis of prognosis. The heterogeneity among the studies was high (*I*^2^ = 69.2%), thus a REM was used for analysis (Table 2). The forest plot is shown in Figure 2. The results showed that high expression of E-cadherin compared with its low expression, for OS, HR = 0.68 (95% CI: 0.64∼0.73, *P* < .05), which was statistically significant. The subgroup analysis of Ethnicity showed that there were significant differences in both Asian population and Caucasian population. Nevertheless, the heterogeneity did not decrease significantly. Funnel plot (Fig. 4) was basically symmetrical. Egger Test showed that *P* > .05, indicating that there was no publication bias.

**Table 2:** The main results of meta-analysis of the correlation between E-cadherin expression and prognosis, pathological features of NSCLC (E-cadherin high expression vs low expression).

Prognosis and pathological features	n	HR	OR	95% CI	*P*	*I* ^2^	*P* for heterogeneity	Model	*P* (Egger)
OS	32	0.68	NA	0.64∼0.73	.000	69.2	.000	REM	.069
Caucasian	6	0.58	NA	0.50∼0.67	.000	78.3	.000	REM	.572
Asian	26	0.72	NA	0.66∼0.77	.000	65.3	.000	REM	.020
Tumor Stage I-III	19	0.64	NA	0.57∼0.71	.000	60.7	.000	REM	.329
DFS/PFS	8	0.54	NA	0.44∼0.67	.000	39.1	.118	FEM	.250
Tumor Grade (Low vs Middle & High differentiation)	15	NA	0.40	0.27∼0.58	.000	65.0	.000	REM	.001
Tumor Stage (III+IV vs I+II)	11	NA	0.54	0.44∼0.66	.000	19.1	.262	FEM	.131
Lymph node metastases (Positive vs Negative)	10	NA	0.49	0.31∼0.77	.002	77.6	.000	REM	.123

Among them, 19 studies reported the results of E-cadherin expression and OS in patients with tumor stage I-III NSCLC, therefore we conducted a subgroup analysis. The results showed that, for OS, HR = 0.64 (95% CI: 0.57∼0.71).

**Figure 3:**
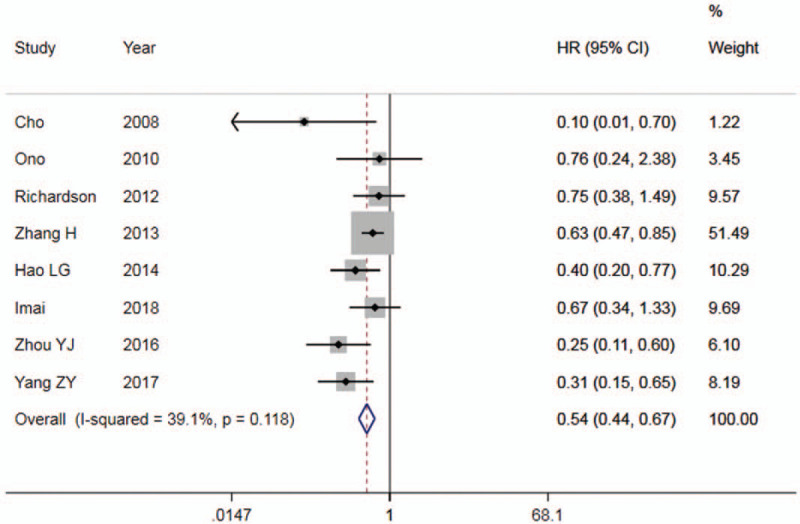
Forest plot of E-cadherin expression and DFS/PFS in patients with NSCLC. DFS = disease-free survival, PFS = progression-free survival, NSCLC = non-small cell lung cancer.

Eight studies^[10,11,18,19,24,39–41]^ reported the relationship between E-cadherin expression and DFS or PFS in patients with NSCLC. The results showed that (Table 2), high E-cadherin expression compared with that of low expression, for DFS or PFS, HR = 0.54 (95%CI: 0.44∼0.67). The forest plot is shown in Figure 3. It was suggested that the patients with high E-cadherin expression had a favorable prognosis in NSCLC.

**Figure 4:**
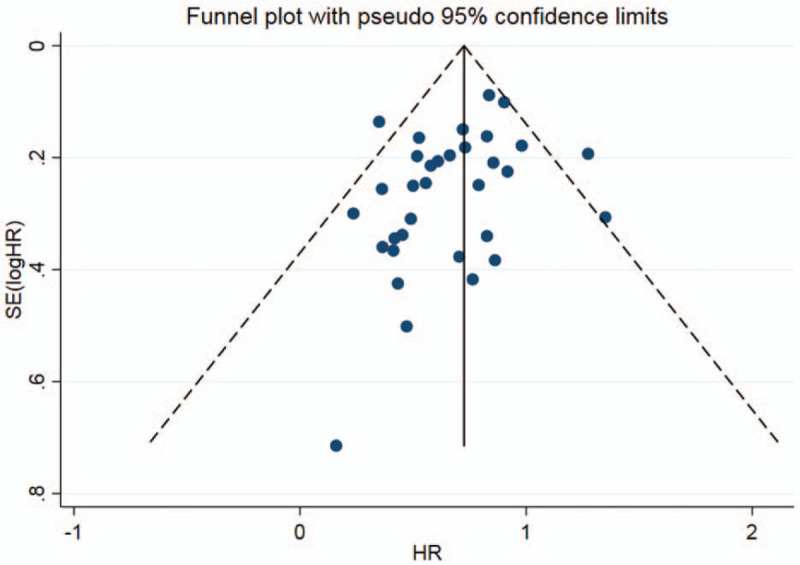
Funnel plot of E-cadherin expression and prognosis in patients with NSCLC. NSCLC = non-small cell lung cancer.

#### 3.2.2 The relationship between expression of E-cadherin and differentiation degree of NSCLC

A total of 15 studies were included,^[7,8,10,11,14,15,19,20,22,23,25–28,33]^ containing 616 patients with low differentiation and 1837 patients with medium and high differentiation. There was mild heterogeneity among studies (*I*^2^ = 65.0%, *P < .*05). Therefore, a REM was used for analysis (Table 2). The results showed that there were notable differences between the 2 groups. The low differentiation compared with the medium and high differentiation, OR = 0.40 (95% CI: 0.27∼0.58, *P* < .01). Egger Test showed certain publication bias (*P* < .05). This indicated that high expression of E-cadherin was able to promote tumor differentiation of NSCLC.

#### 3.2.3 Relationship between E-cadherin expression and NSCLC tumor staging

A total of 11 literatures^[8,10,11,14,19,20,21,25,26,28,29]^ were included, containing 664 patients with tumor stage III+IV and 1337 patients with tumor stage I+II. There was no heterogeneity among studies (*I*^2^ = 19.1%), thus a FEM was performed (Table 2). Tumor stage III+IV compared with tumor stage I+II, OR = 0.54 (95% CI: 0.44∼0.66, *P* < .01). Egger Test showed no publication bias (*P* > .05). This suggested that low expression of E-cadherin presumably promoted tumor staging of NSCLC.

#### 3.2.4 Relationship between E-cadherin expression and NSCLC lymph node metastasis

A total of 10 studies^[8,10,13,14,20,23,25,26,29,35]^ were included, containing 693 patients with lymph node metastasis and 998 patients without lymph node metastasis. There was heterogeneity among studies (*I*^2^ = 77.6%), hence a REM was used for analysis (Table 2). The results showed that high E-cadherin expression compared with that of low expression, OR = 0.49 (95% CI: 0.31∼0.77, *P* < .01). Egger Test showed that there was no publication bias (*P* > .05). It was suggested that the low expression of E-cadherin was likely to promote lymph node metastasis of NSCLC.

### 3.3 Sensitivity analysis

**Figure 5:**
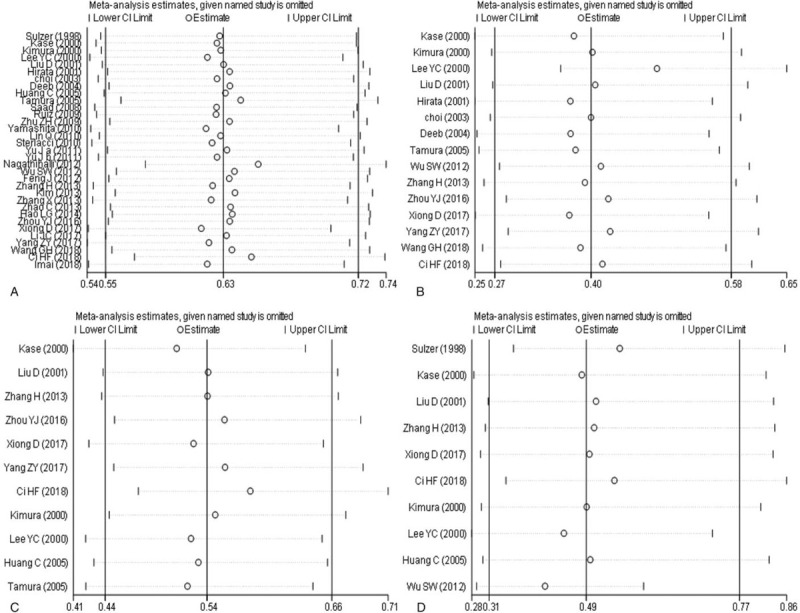
Sensitivity analysis of overexpression of E-cadherin expression and prognosis, pathological features of NSCLC (a: E-cadherin and prognosis; b: E-cadherin and tumor grade; c: E-cadherin and tumor staging; d: E-cadherin and lymph node metastasis). NSCLC = non-small cell lung cancer.

The results of sensitivity analysis are seen in Figure 5. Each study was excluded one by one, and then a meta-analysis was re-performed. The results showed that no significant differences appeared in terms of OS (Fig. 5a), tumor grade (Fig. 5b), tumor stage (Fig. 5c) and lymph node metastasis (Fig. 5d), indicating that the conclusion drawn from this meta-analysis was relatively robust.

## 4 Discussion

In this study, we comprehensively analyzed the correlation between expression of E-cadherin and clinicopathological features of patients with NSCLC, as well as the effect of E-cadherin expression on prognosis of NSCLC. The results showed that expression of E-cadherin was highly related to prognosis of patients with NSCLC, and significantly correlated with tumor stage, differentiation and lymph node metastasis. In the process of occurrence and development of NSCLC, E-cadherin plays an essential role. Zhang et al^[42]^ showed that E-cadherin is the target gene of HOXC8, and deletion of E-cadherin promoted growth and migration of NSCLC. SIX2 is capable of promoting differentiation of non-small cell lung cancer stem cells by transcriptional and epigenetic regulation of E-cadherin.^[43]^ LncRNAs is an important functional regulator involved in progression of non-small cell lung cancer. LncRNA FEZF1-AS1 promotes epithelial-mesenchymal transformation in non-small cell lung cancer by inhibiting E-cadherin and regulating WNT pathway.^[44]^ E-cadherin is critical for the integrity of adhesion junctions between lung epithelial cells. The loss of E-cadherin allows cell movement and is thought to promote the metastasis of lung cancer. Dong et al^[45]^ reported that serum starvation regulates the upregulation of E-cadherin in non-small cell lung cancer A549 cells by activating c-Src.

Meta-analysis of E-cadherin expression and prognosis in NSCLC showed that HR = 0.68 for OS, and HR = 0.54 for DFS or PFS, both of which were statistically significant. It suggested that high expression of E-cadherin is beneficial to prognosis of patients with NSCLC. However, there was severe heterogeneity across studies. In order to explore the source of heterogeneity, we conducted a subgroup analysis of ethnicity and tumor stage. The results showed that for both Caucasian and Asian populations, HR was statistically significant in OS, but heterogeneity did not decrease significantly. Subgroup analysis of patients with stage I-III NSCLC showed that there was a statistically remarkable difference in OS (HR = 0.64), and a considerable decrease in heterogeneity. It showed that tumor staging was an important source of heterogeneity. The funnel plot was basically symmetrical, and *P* value of Egger Test was more than .05, indicating that there was no obvious publication bias. Sensitivity analysis confirmed that expression of E-cadherin was related to prognosis of NSCLC.

Meta-analysis of E-cadherin expression and clinicopathological features of NSCLC showed that low expression of E-cadherin could promote tumor staging and lymph node metastasis and inhibit tumor differentiation. High expression of E-cadherin compared with its low expression, differences were statistically notable in terms of tumor stage, tumor differentiation and lymph node metastasis. From the value of OR, it was observed that there was a strong correlation between E-cadherin expression and tumor stage, differentiation and lymph node metastasis. Sensitivity analysis also confirmed the robustness of the correlation between them. Previous meta-analyses^[46–49]^ showed that low E-cadherin expression is associated with poor prognosis in patients with NSCLC, which is consistent with our research. Qiu et al^[46]^ showed that low expression of E-cadherin promotes lymph node metastasis in patients with NSCLC, which is consistent with the conclusion of this study. However, they suggested that E-cadherin expression is not related to tumor stage and grade of NSCLC patients, which is not consistent with our conclusion. Our study showed that low expression of E-cadherin was likely to promote tumor staging and inhibit tumor differentiation in patients with NSCLC. Yang et al^[49]^ showed that low expression of E-cadherin promotes tumor staging and lymph node metastasis of NSCLC, inhibiting tumor differentiation, which is consistent with our study. Nevertheless, we have included more up-to-date studies, whether in terms of OS or DFS/PFS, or pathological features of NSCLC, which also makes our research more convincing.

Certainly, this study has some limitations. First, our research only included the studies published in English, but not those published in other languages, which might lead to some publication bias. Second, the study of prognosis had significant heterogeneity. Even though subgroup analysis was conducted on the ethnicity of the study population, heterogeneity still existed. In the subgroup analysis of tumor staging, although the heterogeneity decreased, it was only limited to the early and middle stage of NSCLC. For advanced patients, we could not conduct further investigation due to lack of data. Third, the E-cadherin detection methods in the literatures included in this study were IHC, but the study used antibodies different from the first antibody, with different manufacturer and batch number, and the dilution of antibody, which led to potential deviation. Moreover, there were differences in demarcation criteria of E-cadherin expression, which also intended to produce some deviation. Finally, we noted that there was a partial publication bias in tumor differentiation, which might deviate from our conclusions.

In conclusion, our meta-analysis results show that low E-cadherin expression which promotes tumor staging and lymph node metastasis in patients with NSCLC, and inhibits tumor differentiation, is closely related to poor prognosis of patients with NSCLC. Low expression of E-cadherin may be a predictor of poor prognosis in patients with NSCLC. The results of this study also provide a possibility for targeted therapy for patients with NSCLC. However, considering the limitations of this study, more high-quality studies are still needed to verify the correlation between expression of E-cadherin and patients with NSCLC.

## Author contributions

Dong Chao, Qingxin Li: Critical revision of the manuscript; Dong Chao, Qingxin Li: Substantial contribution to the conception and design of the work, manuscript drafting; Dong Chao, Gawei Hu, Qingxin Li: Acquisition, analysis, and interpretation of the data; Dong Chao, Gawei Hu, Qingxin Li: Revising the manuscript critically, final approval of the version to be published. All authors have read and approved the final manuscript.

**Conceptualization:** Dong Chao, Gawei Hu, Qingxin Li.

**Data curation:** Dong Chao, Gawei Hu, Qingxin Li.

**Formal analysis:** Dong Chao, Gawei Hu.

**Funding acquisition:** Dong Chao, Qingxin Li.

**Investigation:** Dong Chao, Gawei Hu, Qingxin Li.

**Methodology:** Dong Chao, Gawei Hu.

**Project administration:** Dong Chao, Gawei Hu, Qingxin Li.

**Resources:** Dong Chao, Gawei Hu, Qingxin Li.

**Software:** Dong Chao, Gawei Hu.

**Supervision:** Dong Chao, Gawei Hu, Qingxin Li.

**Validation:** Dong Chao, Gawei Hu.

**Visualization:** Dong Chao, Gawei Hu.

**Writing – original draft:** Dong Chao, Gawei Hu.

**Writing – review & editing:** Dong Chao, Gawei Hu, Qingxin Li.
